# DNA Barcodes Provide a Quick Preview of Mitochondrial Genome Composition

**DOI:** 10.1371/journal.pone.0000325

**Published:** 2007-03-28

**Authors:** Xiang Jia Min, Donal A. Hickey

**Affiliations:** Department of Biology, Concordia University, Montreal, Quebec, Canada; Ecole Normale Supérieure de Lyon, France

## Abstract

DNA barcodes have achieved prominence as a tool for species-level identifications. Consequently, there is a rapidly growing database of these short sequences from a wide variety of taxa. In this study, we have analyzed the correlation between the nucleotide content of the short DNA barcode sequences and the genomes from which they are derived. Our results show that such short sequences can yield important, and surprisingly accurate, information about the composition of the entire genome. In other words, for unsequenced genomes, the DNA barcodes can provide a quick preview of the whole genome composition.

## Introduction

DNA barcodes are short, standardized sequences of DNA that are used to assign unknown biological specimens to known species [1]–[2]. Because of the rapid growth in the numbers of these sequences, coupled with their very broad taxonomic sampling, they could, potentially, provide insights into patterns of molecular evolution and population genetics [3].

Here, we show that several important attributes of complete mitochondrial genomes can be predicted with high accuracy from the short barcode sequences alone. These attributes include average nucleotide composition, patterns of strand asymmetry, and the high frequency of codons that encode hydrophobic amino acids.

This means that DNA barcodes, or other short sequences sampled from a wide taxonomic range, can give a meaningful overview of variations in genome composition long before complete genome sequences become available for many of the sampled taxa.

## Methods

A total of 849 complete mitochondrial (mt) genome (mtDNA) sequences with detailed gene annotation in GenBank format in metazoa were downloaded from NCBI RefSeq organelle genome database (July, 2006 release) (Supplementary Table S1). The DNA sequences of protein coding genes were extracted from each mtDNA sequence.

DNA barcode sequences were extracted from same 5′ regions of cytochrome c oxidase subunit 1 (COI) genes, as reported by Herbert et al. (2004) [1], based on aligned COI coding DNA sequences. The COI coding DNA sequence alignments were guided by pre-aligned COI protein sequences by using MEGA3 software [4]. The barcode sequences have a length ranged from 624–663 bp (average 648 bp) which contain 208–221 codons (average 216 codons). We wrote a short computer script to calculate nucleotide frequencies.

All the barcode sequences are located on the “+”-strands of mt genomes, except the following three entries: *Spadella cephaloptera* (NC_006386), *Saccoglossus kowalevskii* (NC_007438) and *Lampsilis ornata* (NC_005335). The base frequencies of mtDNA were calculated by using the strand which contains a DNA barcode. The G+C (GC) content and A+T (AT) content of protein coding DNA sequences are calculated by using all protein coding DNA sequences from both strands. The GC and AT asymmetry is measured in terms of GC- and AT-skews according to the following formulae: GC-skew = (G-C)/(G+C); AT-skew = (A-T)/(A+T), where C, G, A, and T are the occurrences of the four nucleotides [5].

## Results and Discussion

We compiled statistics on mitochondrial genome composition based on more than 800 completely-sequenced genomes of animal mitochondria (see Methods). We then compiled these same statistics for the standard DNA barcode region, comprising only 648 base pairs from the 5′ region of the mitochondrial cytochrome oxidase subunit I gene (COI) [1]–[2]. Using these two sets of statistics, we asked how well the short DNA barcode sequences reflected the average properties of the genomes from which they were sampled.

**Figure 1:**
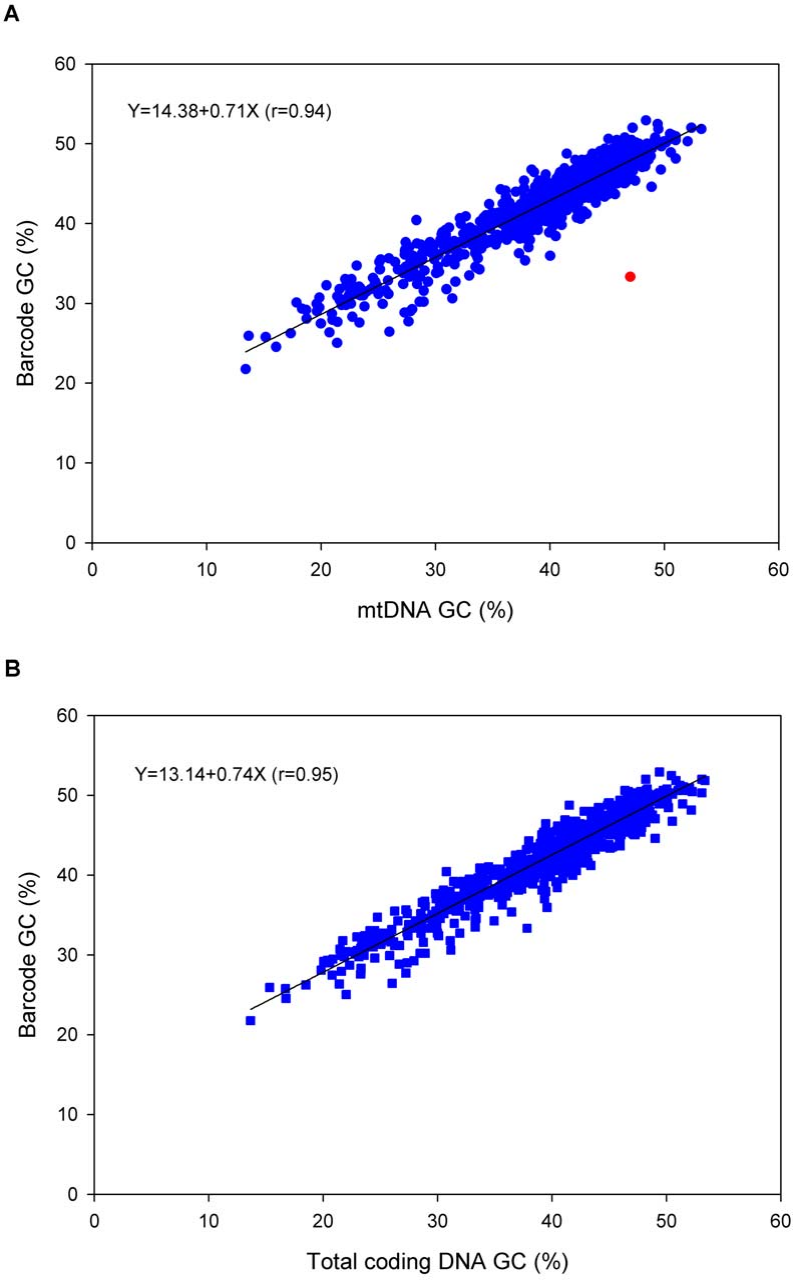
Correlation between nucleotide content of DNA barcodes and the nucleotide content of the mitochondrial genome. Panel A shows the GC content of the DNA barcodes plotted against the GC content of the entire mitochondrial genome (including protein-coding and non-coding sequences). The outlier point (shown in red) is from *Trichoplax adhaerens* (NC_008151). Panel B shows the DNA barcode GC content plotted against the GC content of the combined protein-coding sequences only.

A comparison of the nucleotide composition (GC content) of the DNA barcode sequences with the entire mitochondrial sequences is shown in Fig. 1A. From the Figure, we can see that there is an excellent match between the DNA barcodes and their “parent” genomes, and this relationship is highly significant (correlation coefficient = 0.94). The one outlier point, shown in red, is from *Trichoplax adhaerens* (NC_008151) which has an unusually large mitochondrial genome that, unlike other animal mitochondria, contains a large proportion of non-coding DNA [6]. When we confine the analysis to the mitochondrial coding sequences only (see Fig. 1B) the outlier point disappears and the correlation is even higher (r = 0.95). These results show that the nucleotide composition of the DNA barcodes is an excellent predictor of the composition of the entire mitochondrial genome.

**Figure 2:**
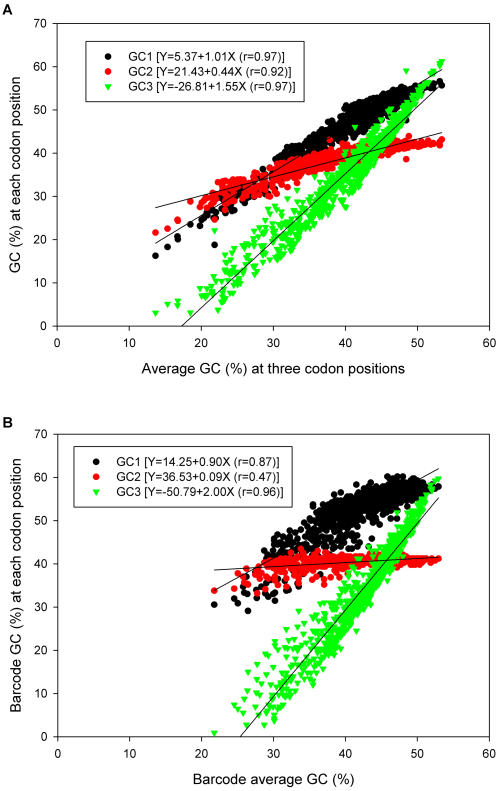
Correlation between GC content at each codon position and the average GC content. Panel A shows the results for all protein coding sequences in the mitochondrial genome. Panel B shows the results based on the DNA barcode region alone.

Variations in GC content affect different codon positions to a greater or lesser degree. Specifically, the third codon position typically shows the largest range of variation and the second codon position shows the least variation. These differences between the codon positions reflect differences in the degree of selective constraint. Fig. 2A shows the correlation between the GC content at each of the three codon positions and overall GC content of mitochondrial coding sequences. The results show the expected pattern of high variation at the third codon position and lowest variation at the second codon position. These same patterns can be seen when we confine the analysis to the DNA barcode sequences alone (see Fig. 2B). Again, the patterns observed in the short barcode sequences reflect very accurately the patterns seen for the entire coding region.

**Figure 3:**
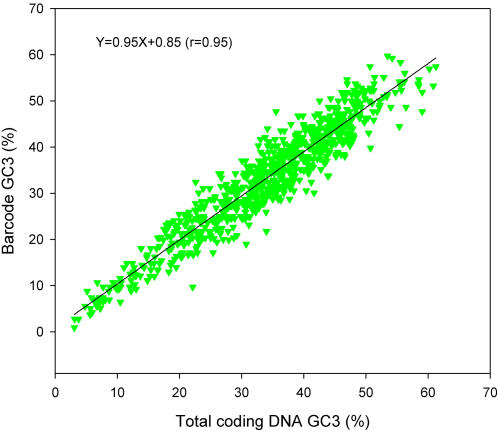
Correlation between GC content of the entire mitochondrial genome and the DNA barcode sequences. In this case, the nucleotide contents were calculated for the third codon position only.

The data presented in Fig. 2A not only illustrate the greater conservation of nucleotide composition at the second codon position, but they also show that mitochondrial genomes have a lowered GC content at the second position, even for those species where the GC content at the other two codon positions is greater than 50%. This lower GC content at the second codon position is a reflection of the selection for T-containing codons (see below). This effect is also seen clearly when we examine the data from the barcode sequences alone (see Fig. 2B). In this case, the slope of the GC2 line is only 0.09, meaning that the GC content at this position is relatively insensitive to the genomic changes in GC content. This is explained by the fact that the barcode region is subject to intense purifying selection at the protein level. If instead, we confine our analysis to the third codon positions of both the barcode and the entire mitochondrial coding region, we avoid the complication of protein-level selective constraint–because the majority of mutational changes at this position are silent at the protein level. The results of such an analysis are shown in Figure 3. As expected, in this case there is an excellent correlation between the GC3 content of the barcode sequence and the GC3 content of the corresponding genome from which it was derived.

**Figure 4:**
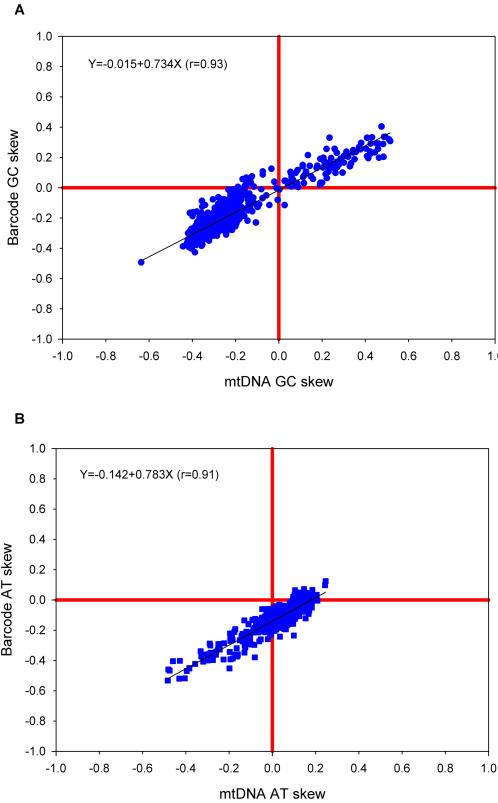
Correlations between nucleotide skews in the entire mitochondrial genome and in the DNA barcode sequences. Panel A shows the results for GC skew [(G-C)/(G+C)]. Panel B shows the results for AT skew [(A-T)/(A+T)].

**Figure 5:**
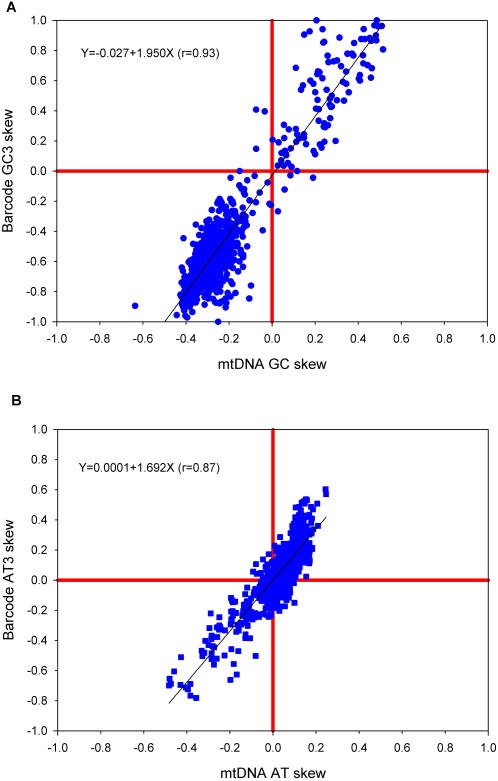
Correlations between nucleotide skews in the entire mitochondrial genome and in the DNA barcode sequences (third codon position only). Panel A shows the results for GC skew [(G-C)/(G+C)]. Panel B shows the results for AT skew [(A-T)/(A+T)].

In addition to variations in GC content, mitochondrial genomes also vary in their patterns of strand asymmetry (usually measured as GC skew and AT skew) [5]. For example, for a given GC base pair in the mammalian mitochondrial genome, the C nucleotide is found more frequently on the coding strand while the complementary G is found on the template strand. This negative GC skew is not, however, shared by all animal taxa. Likewise, there are variations in AT skew between lineages. Again, we asked if these variations between lineages in strand asymmetry might be detectable from the DNA barcode sequences alone. The relationship between the GC skew of the whole mitochondrial genome and that of the DNA barcode region is shown in Figure 4A and we see that there is an excellent correlation (r = 0.93). The same is true for the variations in AT skew (Fig. 4B). In Figure 4A, there is a clustering of points in the lower left quadrant. This reflects the overrepresentation of mammalian sequences in the database. A more interesting trend is seen in Figure 4B. Here, we see that the AT skew extends into the negative range, but there are no large positive values for AT skew. This is because the strand asymmetry is counteracted by strong selection for codons containing T at the second codon position, which encode hydrophobic amino acids [7]. These amino acids are strongly selected for in mitochondrial genomes because mitochondrial proteins have multiple membrane-spanning domains. The effect is especially strong in the barcode sequences because the barcode sequences are from a region the COI gene that encodes membrane spanning domains. As a control, we can look at the patterns of AT skew that occur at the third codon position, where this selection in favor of hydrophobic amino acids would not be operative. The results for the third codon position (see Fig. 5) confirm this prediction. In that case, there is no countervailing selection and we see positive values for AT skew.

### Conclusion

The results presented here illustrate a principle that would apply not only to DNA barcode sequences, but to any form of strategic sampling of short sequences from larger genomes. Just as DNA barcodes and metagenomic studies can provide a snapshot of species-specific sequence variations, surprisingly small sequence samples can also provide an accurate reflection of genome composition.

## Supporting Information

Table S1 The names of the species used in this study, along with the GenBank accession numbers of their mitochondrial genome sequences are provided in the Supplementary Table S1.
(0.11 MB XLS)
